# Pattern Switchable Antenna System Using Inkjet-Printed Directional Bow-Tie for Bi-Direction Sensing Applications

**DOI:** 10.3390/s151229851

**Published:** 2015-12-10

**Authors:** Seung-Hyun Eom, Yunsik Seo, Sungjoon Lim

**Affiliations:** Electrical and Electronics Engineering, Chung-Ang University, 84 Heukseok-ro, Dongjak-gu, Seoul 06974, Korea; umsh0303@gmail.com (S.-H.E.); bampiresys@nate.com (Y.S.)

**Keywords:** bow-tie antenna, inkjet-printing technology, paper-based antenna, pattern switchable antenna, reconfigurable antenna

## Abstract

In this paper, we propose a paper-based pattern switchable antenna system using inkjet-printing technology for bi-direction sensor applications. The proposed antenna system is composed of two directional bow-tie antennas and a switching network. The switching network consists of a single-pole-double-throw (SPDT) switch and a balun element. A double-sided parallel-strip line (DSPSL) is employed to convert the unbalanced microstrip mode to the balanced strip mode. Two directional bow-tie antennas have different radiation patterns because of the different orientation of the reflectors and antennas. It is demonstrated from electromagnetic (EM) simulation and measurement that the radiation patterns of the proposed antenna are successfully switched by the SPDT switch.

## 1. Introduction

Reconfigurable antennas have attracted attention because of their potential applications and advantages. There are many types of reconfigurable antennas: radiation pattern, frequency, polarization, and combined antennas. Especially, radiation pattern reconfigurable antennas can overcome noisy environments, improve security, prevent from electronic jamming, and save energy [1]. Up to now, pattern reconfigurable antennas have been realized using various switching component such as PIN diodes, varactor diodes, MEMS (micro-electro-mechanical systems) switches, and FET (field effect transistor) components [2,3,4,5,6].

In addition, there is a growing interest in easy-to-use inkjet-printing technology to manufacture electronics on flexible substrates. Inkjet printing is a noncontact printing technology. It becomes possible to print patterns on a substrate directly with the droplets ejected from the nozzle. Compared with inkjet-printing technology, conventional photolithography technology has a high exposure apparatus cost, a complex process, environmental pollution, and large material losses that accompany the process [7]. A direct printing process such as inkjet-printing technology consists only of the printing process and the sintering process. Therefore, a complicated and long-duration process can be replaced by a cost-effective and simple process. With inkjet-printing technology, there is no unnecessary loss of materials. Flexible electronics can be inkjet-printed on flexible substrates such as polyethylene, terephthalate, polyimide, and paper [8,9,10]. Inkjet-printing technology has been used in various RF applications such as the inkjet-printed radio-frequency identification (RFID) tag [11], ultrawideband antennas [12], gas-detection sensor [13], high frequency inductors and capacitors [14], frequency selective surfaces (FSS) [15], composite right/left-handed transmission line [16], flexible metamaterial absorber [17], terahertz split ring resonator [18] and artificial magnetic conductor (AMC) structure [19].

In this study, a paper-based pattern switchable antenna is proposed using inkjet-printing technology. The proposed antenna system is composed of two directional bow-tie antennas and a switching network. The switching network is built on a printed-circuit-board (PCB) and consists of a balun element and an SPDT switch. The bow-tie antennas with two different radiation patterns are printed on paper, and a reflector is added on the same paper to enhance the directivity. Because of two different radiation patters, the proposed antenna system is useful for bi-direction sensor applications. The final antenna system is realized by bonding the inkjet-printed antennas on paper and the switching network on the PCB. In order to achieve low-cost fabrication, we used a commercial inkjet printer that is commonly used at home, instead of an expensive material printer that is usually used in industry.

## 2. Antenna System Design

### 2.1. Directional Bow-Tie Antenna Design

**Figure 1:**
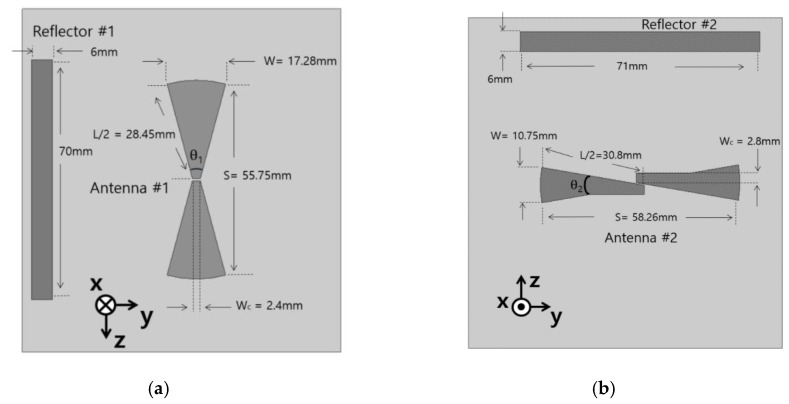
Layout of two inkjet-printed bow-tie antennas. (**a**) first antenna and (**b**) second antenna.

Figure 1 illustrates two directional bow-tie antennas. First, an omnidirectional bow-tie antenna is designed to resonate at 1.8 GHz. Its parameters are determined by Equation (1)–(5) [20,21]:

(1) $$f_{r}=\frac{c}{2\sqrt{\varepsilon_{e}}}(\frac{1.152}{R_{t}})$$

(2) $$R_{t}=\frac{L}{2}\frac{\left(W+2\Delta l)+(W_{c}+2\Delta l\right)}{\left(W+2\Delta l)(S+2\Delta l\right)}$$

(3) $$\Delta l=h\frac{0.412(\varepsilon_{e}+0.3)(\frac{W_{i}}{h}+0.262)}{\left(\varepsilon_{e}-0.258)(\frac{W_{i}}{h}+0.813\right)}$$

(4) $$\varepsilon_{e}=(\frac{\varepsilon_{r}+1}{2})+(\frac{\varepsilon_{r}-1}{2}){\left(1+\frac{12h}{W_{i}}\right)}^{-1/2}$$

(5) $$W_{i}=(\frac{W+W_{c}}{2})$$

**Table 1:** Dimension of Bow-Tie Antennas.

	W	S	L/2	W_c_
Antenna #1	17.28 mm	55.75 mm	28.45 mm	2.4 mm
Antenna #2	10.75 mm	58.26 mm	30.8 mm	2.8 mm

**Figure 2:**
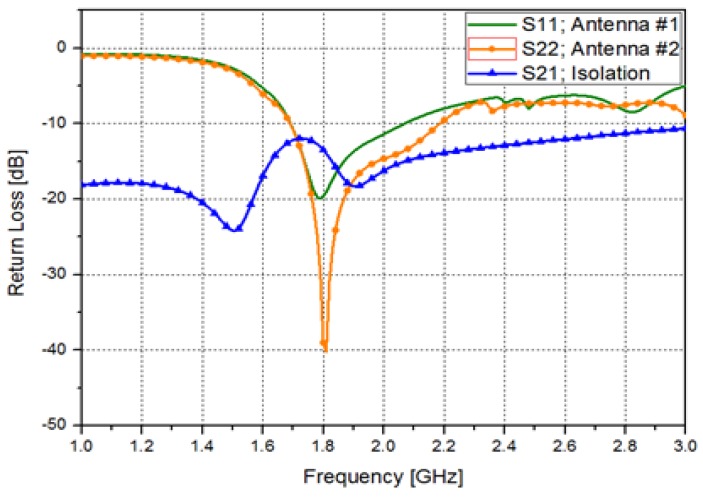
Simulated S-parameters of the designed antennas; return loss of the first antenna (S_11_), return loss of the second antenna (S_22_), and isolation between two antennas (S_21_).

The relative permittivity, effective permittivity, and thickness of the substrate are denoted by εr, εe, and h. The other geometrical parameters are defined in Figure 1 and Table 1. For pattern-switching capability, a directional antenna is preferred. Because a bow-tie antenna has an omnidirectional radiation pattern, a directional bow-tie antenna is designed by loading a reflector as shown in Figure 1 [22,23]. In addition, two directional bow-tie antennas are arranged orthogonally to each other for high isolation. We determined the optimum position, length, and width of the reflector to achieve highest directivity. Its performances are simulated by ANSYS high frequency structure simulator (HFSS). Figure 2 shows the simulated S-parameters of the antennas #1 and #2. At 1.8 GHz, the simulated return losses of the antenna #1 and #2 are 19.62 dB and 38.99 dB, respectively. The isolation between two antennas (S_21_) is −13.49 dB at 1.8 GHz. The simulated 10-dB impedance bandwidths of the antenna #1 and #2 are 1.69–2.07 GHz and 1.69–2.19 GHz, respectively. The bandwidth of each antenna is controlled by the width Wc [24].

**Figure 3:**
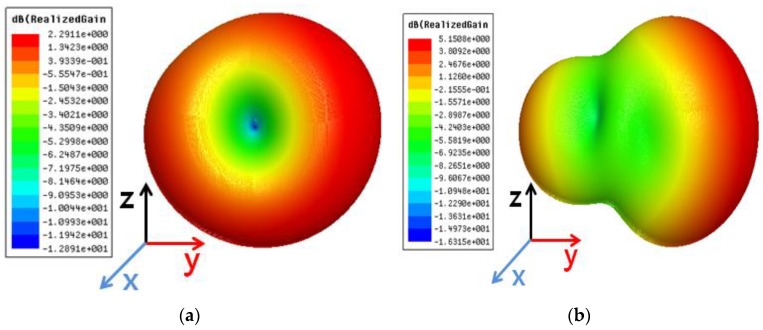

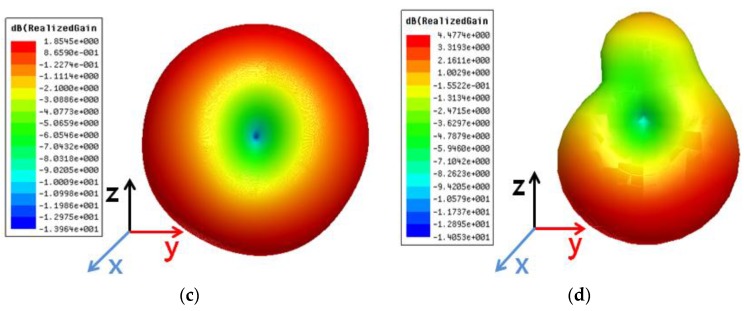
Simulated 3D radiation patterns when (**a**) first antenna is on without reflector, (**b**) first antenna is on with reflector, (**c**) second antenna is on without reflector and (**d**) second antenna is on with reflector.

Figure 3a,b show the simulated 3D radiation patterns of the antenna #1 without and with the reflector, respectively. Due to the reflector, the peak gain is increased from 1.85 dBi to 4.47 dBi. Figure 3c,d show the simulated 3D radiation patterns of the antenna #2 without and with the reflector, respectively. Due to the reflector, the peak gain is increased from 2.29 dBi to 5.15 dBi. As shown in Figure 3b,d, the maximum radiation direction of the antenna #1 and #2 are orthogonal each other. Therefore, it is expected that the radiation pattern can be switched by selecting one of two antennas. The radiation efficiencies of the antenna #1 and the antenna #2 are 88.49% and 87.5%, respectively.

### 2.2. Switching Network Design

In order to select one of two directional antennas, the switching network is designed. Because the bow-tie antennas are fed by a balanced signal, a balun is required in the switching network. In this work, a double-sided parallel-strip line (DSPSL) is introduced to transform from unbalanced signal of the microstrip line to balanced signal.

**Figure 4:**
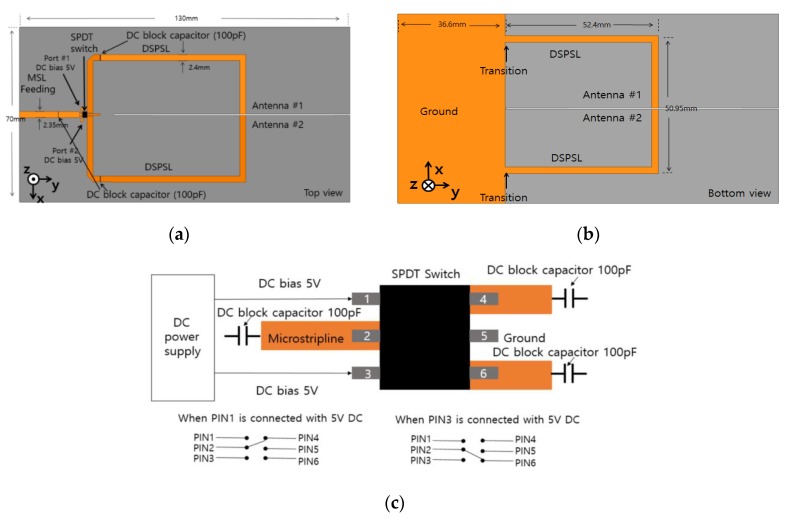
Layout of switching network; (**a**) top view and (**b**) bottom view (**c**) single-pole-double-throw (SPDT) switch with bias network.

The geometrical parameters of the switching network which consists of the DSPSL balun and SPDT switch are indicated in Figure 4. The 50-ohm microstrip line width is 2.35 mm, and the DSPSL line width is 2.4 mm. When the height of the substrate is the same, the DSPSL has a higher characteristic impedance than the microstrip line. Therefore, the DSPSL line width is greater than the microstrip line width.

In this work, AS193-73LF (Skyworks Solution, Inc.) is used as the SPDT switch which select the antenna #1 or #2. Figure 4c shows the layout of the SPDT switch with the bias network where three DC blocking capacitors with 100 pF are used at PIN2, PIN4, and PIN6. When a 5 V DC is connected to PIN1, as a result PIN2 is connected to PIN4. Similarly, when a 5 V DC is applied to PIN3, PIN2 is connected to PIN6. The insertion loss and isolation of the SPDT switch are typically 0.45 dB and 19 dB at 1.0–2.0 GHz. The return loss of the SPDT switch is typically 17.7 dB at 1.0–2.5 GHz. For full-wave simulation, the SPDT switch is represented by an equivalent circuit model. In the ON state, the SPDT switch is represented as a series resistance (R_s_ = 1.5 ohm) and a series parasitic inductance (L_s_ = 0.02 nH), In the OFF state, the SPDT switch is represented as a shunt capacitance (C_t_ = 0.015 pF) with a shunt resistance (R_p_) and a series parasitic inductance (L_s_ = 0.02 nH). However, R_p_ of the OFF state can be neglected because resistance is greater than the reactance of C_t_.

**Figure 5:**
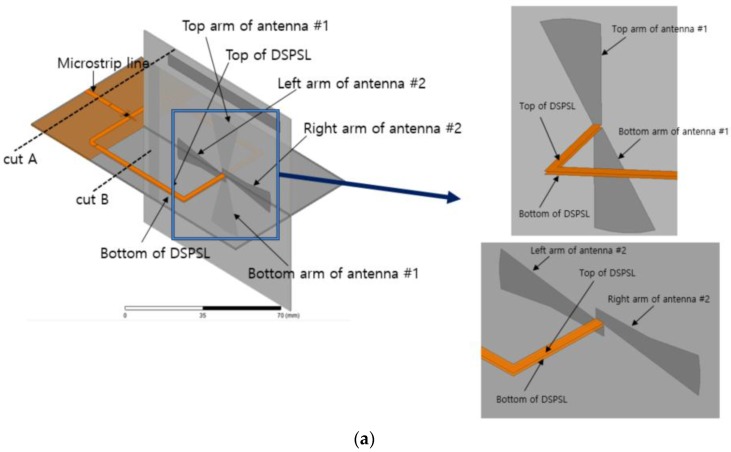

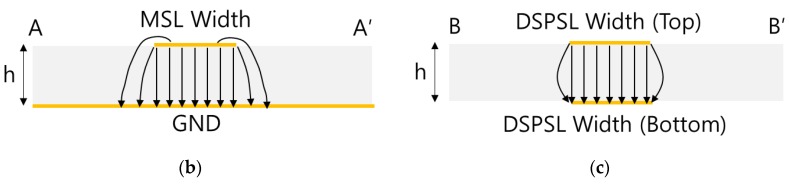
(**a**) 3D-view of antenna with switching network; (**b**) E-field distribution of cut A plane (microstrip line); (**c**) E-field distribution of cut B plane (double-sided parallel-strip transmission line).

Figure 5a shows a three-dimensional view of the proposed antenna with the switching network. As shown in the inset of Figure 5a, the top line of the DSPSL #2 is connected to the left arm of antenna #2 and the bottom line of the DSPSL is connected to the right arm of the antenna #2. Similarly, the top line of the DSPSL #1 is connected to the left arm of antenna #1 and the bottom line of the DSPSL #1 is connected to the right arm of the antenna #1. A microstrip line is an unbalanced line and a DSPSL is a balanced line. Figure 5b,c shows the electric field distributions of the microstrip line and DSPSL, respectively. When the perfect electric conductor is placed at the center of the substrate and parallel to the strips, the DSPSL is identical to two back-to-back placed microstrip lines. Thus, it can be easily analyzed using image theory [25,26,27]. At the DSPSL output (B-B′ plane), out-of-phase signals are combined while the in-phase signals are cancelled out. In addition, the DSPSL has wider linewidth compared to a microstrip line with the same characteristic impedance because the guided wavelength of the DSPSL is lower than that of the microstrip line.

## 3. Fabrication and Measurements

**Figure 6:**
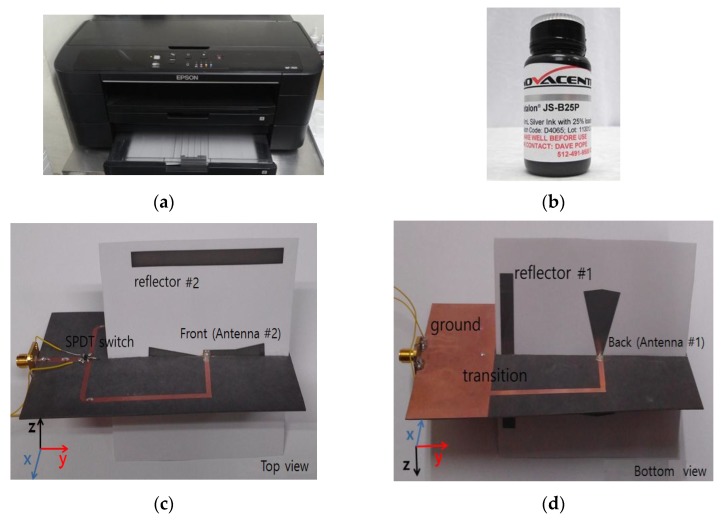
Pictures of home inkjet printer, silver nanoparticle ink and fabricated antenna system; (**a**) home printer (WF-7011); (**b**) silver ink (JS-B25); (**c**) prototype antenna top view; and (**d**) prototype antenna bottom view.

Two directional bow-tie antennas are inkjet-printed on photo paper using a home printer (Epson WF-7011) which is shown in Figure 6a. We used silver nanoparticle ink (JS-B25P, Novacentrix) for printing which contains 25% silver as shown in Figure 6b. The sheet resistance of the silver nanoparticle ink is 60 mΩ. To increase the conductivity, sintering process is necessary. At lower temperature, large interval exists between the particles. At higher temperature, gap of the particles start to diminish [11,22]. The photo paper can withstand a temperature of 180 °C. Therefore, a sintering process at a temperature of 180 °C was used.

The proposed bow-tie antennas are inkjet-printed separately, one on each of the two photo papers. Next, two inkjet-printed bow-tie antennas are bonded together in such a way that one antenna remains on the top and the other remains on the bottom side. A nonconductive adhesive material is used to bond them. The switching network was fabricated on a substrate of Duroid-5870 having a dielectric constant of 2.33, a loss tangent of 0.0012, and a thickness of 0.78 mm.

Nonconductive epoxy is used to bond the switching network on the Duroid-5870 and the inkjet-printed antennas on photo paper. Conductive silver epoxy (CW2400) is used to bond the signal lines. The volume resistivity of CW2400 is less than 0.001 ohm-cm; therefore, it has high enough conductivity to transmit signals. Figure 6c,d show the pictures of top and bottom view of the fabricated antenna system prototype.

**Figure 7:**
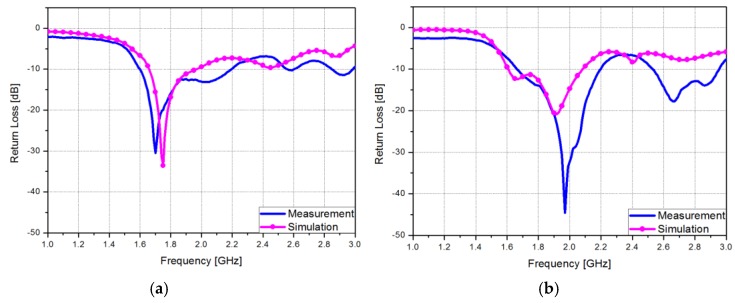
Simulated and measured return losses of the proposed antenna system when (**a**) first antenna is on and (**b**) second antenna is on.

A vector network analyzer was used for measurement of the fabricated antenna system. Figure 7 shows simulated and measured return loss of the proposed antenna system. When the antenna #1 is selected, the measured 10 dB impedance bandwidth is 1.61–2.22 GHz. When the antenna #2 is selected, the measured 10 dB impedance bandwidth is 1.66–2.2 GHz. Both modes provide a 10 dB impedance bandwidth of approximately 600 MHz. It can be confirmed that look similar results compared to the simulated results. When the antenna #2 is on, the resonance frequency was considerably shifted.

**Figure 8:**
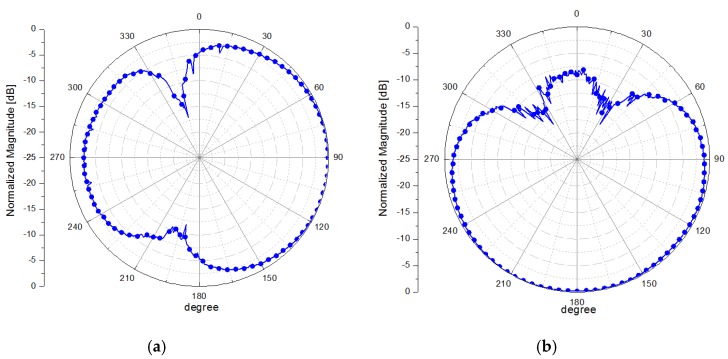
Measured E-plane radiation patterns of fabricated antenna when (**a**) first antenna is on and (**b**) second antenna is on.

The radiation pattern of the proposed antenna system is measured in an anechoic chamber. Figure 8 shows the normalized results of the radiation patterns obtained by the measurement when antenna #1 is on and antenna #2 is off, and vice versa. It is successfully demonstrated that the maximum radiation direction can be switched. The measured peak gains are 3.92 dBi and 4.72 dBi when antenna #1 and antenna #2 are selected, respectively.

## 4. Conclusions

In this paper, we proposed a paper-based pattern switchable antenna using inkjet technology. Two directional bow-tie antennas with different radiation patterns are inkjet-printed on paper. In both radiation modes, the 10 dB impedance bandwidth is in the range 1.66–2.2 GHz. When antenna #1 and antenna #2 are selected, the peak gains are 3.96 and 4.72 dBi, respectively. It is numerically and experimentally demonstrated that two different radiation patterns are successfully switched by using the SPDT switch. Therefore, the proposed pattern switchable antenna can sense the objects at different location. In the present prototype, the switching network is fabricated on the PCB and the antennas are inkjet-printed on paper. This hybrid structure requires mechanical assembly processes. As future works, all-inkjet-printed prototype will be built by inkjet-printing the switching network on paper.
